# COPII vesicles and the expansion of the phagophore

**DOI:** 10.7554/eLife.44944

**Published:** 2019-01-29

**Authors:** Catherine Rabouille

**Affiliations:** 1 Hubrecht InstituteRoyal Netherlands Academy of Arts and Sciences and University Medical CenterUtrechtThe Netherlands; 2 Biomedical Science of Cells and SystemsUniversity Medical Center GroningenGroningenThe Netherlands

**Keywords:** human, autophagy, cell, phagophore, COPII vesicles, ERGIC, Human

## Abstract

A new study has identified the proteins that adapt COPII vesicles to the needs of starving cells.

**Related research article** Jeong YT, Simoneschi D, Keegan S, Melville D, Adler NS, Saraf A, FlorensL, Washburn MP, Cavasotto CN, Fenyö D, Cuervo AM, Rossi M, Pagano M. 2018. The ULK1-FBXW5-SEC23B nexus controls autophagy. *eLife* **7**:e42253. doi: 10.7554/eLife.42253

When a cell is starving, it can recycle cytoplasmic elements that are dispensable or faulty to obtain the amino acids and nutrients it needs to survive. This process, known as autophagy, is orchestrated by more than 40 different proteins (Mizushima et al., 2011). It starts with the formation of the phagophore, a flat membrane-bound structure that expands and engulfs the components destined for digestion. Although the membrane of many organelles – including the Golgi apparatus – can be the source of the phagophore, the structure appears to largely emerge from the endoplasmic reticulum (ER). It has recently also become clear that the early expansion of the phagophore requires vesicles coated with a protein complex called COPII, hereafter referred to as COPII vesicles (reviewed in van Leeuwen et al., 2018).

Cells with plenty of nutrients grow by producing proteins, and COPII vesicles are essential in this process. They bud from ER exit sites and transport newly synthesized proteins to the Golgi and many other membrane compartments. An array of molecules works together on the membrane at the ER exit sites to form these vesicles. Indeed, the transmembrane ER protein Sec12 and the small GTPase Sar1 help to recruit the Sec23/Sec24 complex that will create the inner coat of the vesicle, while another complex, Sec13/31, will then form the outer coat (Gomez-Navarro and Miller, 2016). However, while starving cells require COPII vesicles for autophagy, cells deprived of nutrients reduce their protein secretion (Jeong et al., 2018; Zacharogianni et al., 2014): in this context, how can these vesicles be specifically generated to help expand the phagophore?

Several complementary models have been put forward to explain how this could be possible. Some propose that once the COPII vesicles have formed at the ER exit sites, they are redirected away from the Golgi and towards the nascent phagophore (Rao et al., 2016; Tan et al., 2013; Davis et al., 2016; Wang et al., 2015). Another model suggests that in starving mammalian cells, instead of being redirected, COPII vesicles bud from a different place altogether. Indeed, during starvation, COPII vesicles emerge from a membrane between the ER and the Golgi, the ER-Golgi intermediate compartment (ERGIC). Autophagy activates an enzyme, the kinase ULK1, which drives this change by helping to relocate the COPII protein Sec12 to the ERGIC (Ge et al., 2017). Once the vesicles have budded from this structure, they go on to expand the phagophore (Ge et al., 2015). Now, in eLife, Michele Pagano and colleagues at New York University School of Medicine, IBioBA in Buenos Aires and other institutes in the United States and Argentina – including Yeon-Tae Jeong as first author – report results that support and expand this last model (Jeong et al., 2018).

Combining state-of-the-art biochemistry with imaging, Jeong et al. show that COPII vesicles do indeed bud from the ERGIC during autophagy. But the experiments also reveal that the inner coat of these vesicles is different. When cells are growing, an enzyme known as FBXW5 tags Sec23B for destruction, and the protein is then degraded slowly, but constantly. Under these conditions, the remaining Sec23B proteins may bind Sec24 and help form COPII vesicles destined for secretion, but this is likely a small contribution.

The study by Jeong et al. further reveals that when starvation activates ULK1, the enzyme then phosphorylates Sec23B to produce Sec23B-P, which is impervious to FBXW5; Sec23B-P starts to accumulate and bind to Sec24A and Sec24B – but not Sec24C or Sec24D. The Sec23B-P/Sec24AB complex then relocates to the ERGIC (possibly as a direct consequence of the phosphorylation of Sec23B), and it forms COPII vesicles specifically destined to fuel the growth of the phagophore. The beauty of these experiments is thus to show that certain COPII subunits have a clear, dedicated role during autophagy, with Sec23B-P/Sec24AB playing a key part in forming the vesicles required in this process (Figure 1). It remains to be examined whether Sec23B-P/Sec24AB also helps transport proteins to the Golgi under starvation conditions, when general secretion is reduced and the complex is efficiently brought to the ERGIC. While it is likely that the complex is specific to autophagy, this would need to be further investigated.

**Figure 1.**
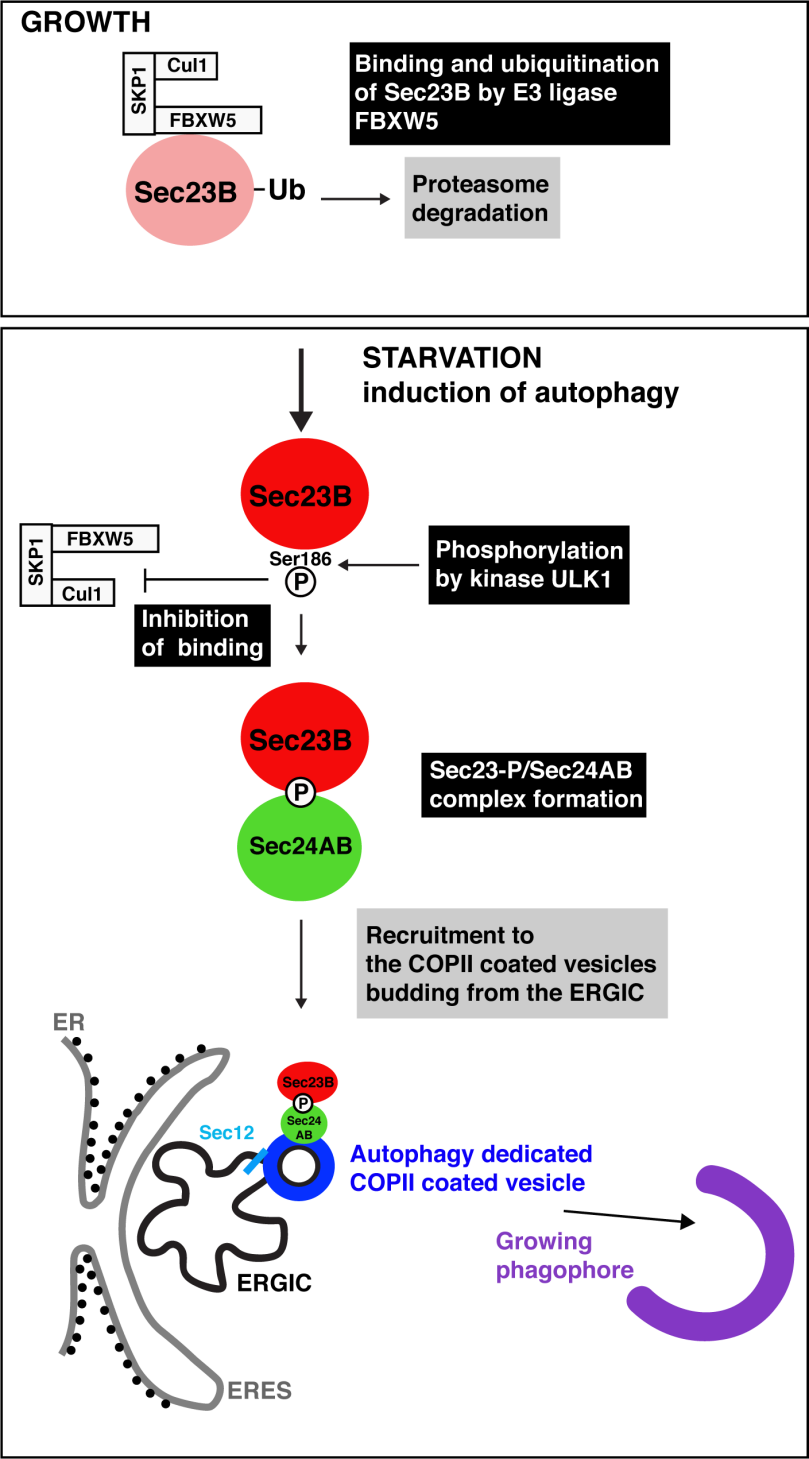
Autophagy starts with the production of special COPII vesicles. When cells are growing (top), the E3 ligase FBXW5, which is associated with Cul1 and SKP1, tags Sec23B (pink circle) with ubiquitin (Ub). This labels Sec23B for destruction by the cell. Starvation kick-starts autophagy, whereby the cell recycles certain components in order to obtain amino acids and nutrients. It also activates ULK1, a kinase that phosphorylates Sec23B (red circle) on Serine 186, thus preventing FBXW5 from tagging it for destruction. Instead, Sec23B-P associates with Sec24AB (green) to form a complex that is not recruited to the endoplasmic reticulum exit sites (ERES; gray line), but to the ER-Golgi intermediate compartment (ERGIC; black line). Another COPII protein, Sec12 (light blue), has also been relocated to this structure. This creates special COPII vesicles (dark blue) that bud to fuel the growth of the phagophore (violet structure) and autophagic activity.

The work by Jeong et al. also helps to better grasp how ULK1 controls the use of COPII proteins during autophagy: the kinase helps to relocate the machinery from ER exit sites to the ERGIC, while also tweaking the nature of the vesicles’ inner coat to fuel the early expansion of the phagophore. These results add to work at Berkeley, which showed how ULK1 participates in bringing Sec12 to the ERGIC, initiating the formation of COPII vesicles (Ge et al., 2015; Ge et al., 2017). In yeast, ULK1 also contributes to the recruitment of a protein that, once activated, will direct COPII vesicles to the phagophore assembly site (Wang et al., 2013). Next, it will be interesting to learn how the enzyme acts on other components of the ER exit sites – including Sec16, a ULK1 substrate during periods of growth (Joo et al., 2016).
